# Kinematic Alignment of Failed Mechanically Aligned Total Knee Arthroplasty Restored Constitutional Limb Alignment and Improved Clinical Outcomes: A Case Report of 7 Patients

**DOI:** 10.3390/jpm12111780

**Published:** 2022-10-28

**Authors:** Elliot Sappey-Mariner, Scott A. Wu, Stefano A. Bini

**Affiliations:** 1 Department of Orthopaedic Surgery, University of California, San Francisco, CA 94143, USA; 2 Feinberg School of Medicine, Northwestern University, Chicago, IL 60611, USA

**Keywords:** total knee arthroplasty, revision total knee arthroplasty, kinematic alignment, failed mechanical alignment, mid-flexion instability, stiffness, constitutional limb alignment

## Abstract

Background: Stiffness and mid-flexion instability (MFI) is a recognized complication of mechanically aligned (MA) total knee arthroplasty (TKA). Kinematic alignment (KA) has been proposed as a means by which to restore normal joint motion following TKA and potentially avoid stiffness and MFI. Several studies have documented improved function with KA when compared to MA. The aim of this study was to determine if revising MA TKAs failed for either MFI or stiffness into KA resolves MFI, achieves better range of motion, and improves clinical outcomes. Methods: A retrospective, single surgeon review was performed. All consecutive TKAs revised from MA into KA for MFI (def: >5 mm opening between 10° and 45° of flexion) or stiffness (def: flexion ≤90°) between January 2017 and May 2021 were included. The constitutional limb alignment of the operated knee was “reverse engineered” by measuring the coronal alignment of the contralateral healthy knee or pre-operative x-rays. Femoral Rotation was set at 3 degrees internal to the trans epicondylar axis. All coronal and sagittal angles were digitally measured on pre- and post-operative long leg and maximum flexion radiographs (minimum 12 month follow-up). The Knee Society Score (KSS) and range of motion assessments were collected preoperatively and at final follow-up. Comparisons between groups were done with a paired T test. Significance was set at *p* < 0.05. Results: Seven patients were included. Two were male, the mean age was 70.1 years (±9.3), mean follow-up was 32 months (±26). Three patients were revised for MFI and 4 for stiffness. Constitutional limb alignment was restored within 2 degrees for all patients. The mean total KSS gain was 65.9 (±18.1). The total KSS was significantly improved in all patients (*p* < 0.001). The mean maximum flexion gain was 30 deg (±23°) (*p* = 0.01). MFI was absent in all patients. Conclusion: In a limited series of patients, revision of stiff or unstable TKA from MA to KA resulted in improved range of motion by 30° on averages, resolved instability without the use of constrained liners, improved clinical outcomes with a mean gain of 75 points on the KSS, and restored constitutional limb alignment within 2 degrees in all patients. As these short term results are promising, further study is warranted.

## 1. Introduction

Recent studies have found that the etiologies for revision total knee arthroplasty (rTKA) are infection, aseptic loosening, instability and stiffness [1]. In particular, rTKA for stiffness or instability is a complex procedure and often results in unreliable clinical outcomes with a high rate of complications including recurrence of stiffness and inferior survival rates compared to primary TKA [2,3].

Mechanical alignment (MA) for primary TKA remains the most common alignment approach [4]. Good to excellent outcomes and excellent survivorship are reported for MA [5]. However, across time, continents and surgeons, 15–20% of the patients remain unsatisfied by their procedure [6]. Symptoms of pain, instability and stiffness are reported by up to 50% of patients [7,8]. 

Stiffness in MA knees is multifactorial. An important predisposition specific to MA may be the fact that “balanced gap” techniques externally rotate the femoral component relative to the posterior condylar axis by approximately 3°. So doing closes down the native lateral flexion gap at 90° which allows the knee to roll back and flex while pivoting around a relatively stable medial “pivot” which remains relatively tight and “balanced” throughout the arc of motion. By closing the lateral flexion gap required for roll back, medial pivot and flexion, the knee inevitably loses flexion [9]. 

Mid-flexion instability (MFI) in MA may be caused by the fact that the components are not aligned with the anatomic axis of rotation of the knee (Rotational Axis) but rather the trans epicondylar axis which has been shown to be 2–5° off axis. In extension, where the knee can be balanced through soft tissue releases because the true and false axes are coplanar, the implant is stable. It is only when the knee is bending and the rotational axes are no longer coplanar that MFI arises and the knee becomes unstable [9]. 

In an effort to improve the clinical results seen with MA TKA, kinematic alignment (KA) has been proposed as an alternative strategy for implant positioning [10]. KA aims to restore the pre-arthritic anatomy of the knee regardless of native alignment relative to the mechanical axis and thereby respect the native soft tissue envelope, soft tissue gaps, and rotational axes [11]. 

The proponents of KA argue that so doing should avoid mid-flexion instability (MFI) and stiffness. To date, KA has shown similar or better clinical outcomes compared to MA at mid-term follow-up for primary TKA [12,13]. Hence, the authors hypothesize that MA TKAs that fail due to stiffness and/or MFI are unlikely to improve if revised back into MA but should improve significantly if revised back to their native alignment using KA principles. The best candidates for a KA rTKA would therefore be those patients whose coronal or sagittal alignment was sufficiently changed from their pre-arthritic anatomy that they develop instability during mid flexion or stiffness due to posterolateral conflict.

One study reported applying restricted KA principles to rTKA [14]. The authors allowed a limited amount of constitutional varus (<4°) in the reconstruction. Good results were reported though in this paper surgical indications included multiple aetiologies (aseptic loosening, implant malposition, infection, instability, anterior compartment overstuffing, patella instability, etc.) and not just stiffness or instability. Thus, no other study was found assessing the use of KA principles specifically to revise MA TKAs that had failed due to these causes. The aim of this study was to report if revising MA TKA patients with MFI or stiffness into KA will restore native limb alignment, resolve MFI, improve range of motion, and impact patient-reported outcomes.

## 2. Materials and Methods

### 2.1. Patients

The scientific and ethics review committees approved the study (Institutional Review Board approval number 19-27178). A single surgeon (S.A.B) retrospective review was performed of all consecutive failed MA TKAs revised into KA for MFI or stiffness between January 2017 and July 2021. For inclusion in this study, the failed index TKA had to have been performed in MA with a final HKA angle of 0° (+/−3°) a femoral joint line angle (FJLA) of 4–6° of valgus and an MPTA angle of 0 degrees (+/−3°). Moreover, the patient’s post-operative radiographic alignment angles had to vary by more than three degrees from their native anatomy for any component or limb alignment angle. Excluded from this review were patients who did not meet the above criteria or were revised for any other etiology (such as infection, aseptic loosening, femoro-tibial instability, patella issues, etc.) even if they were revised into KA. 

### 2.2. Methods of Assessment

Clinical: Function prior to surgery and outcomes following surgery were assessed through chart review at a minimum one year follow up. The Patient Reported Outcome Measure used was the Knee Society Score [15]. The pain was assessed using the visual analog scale (VAS). Patient satisfaction and complication rates were assessed at the last follow-up. 

**Figure 1:**
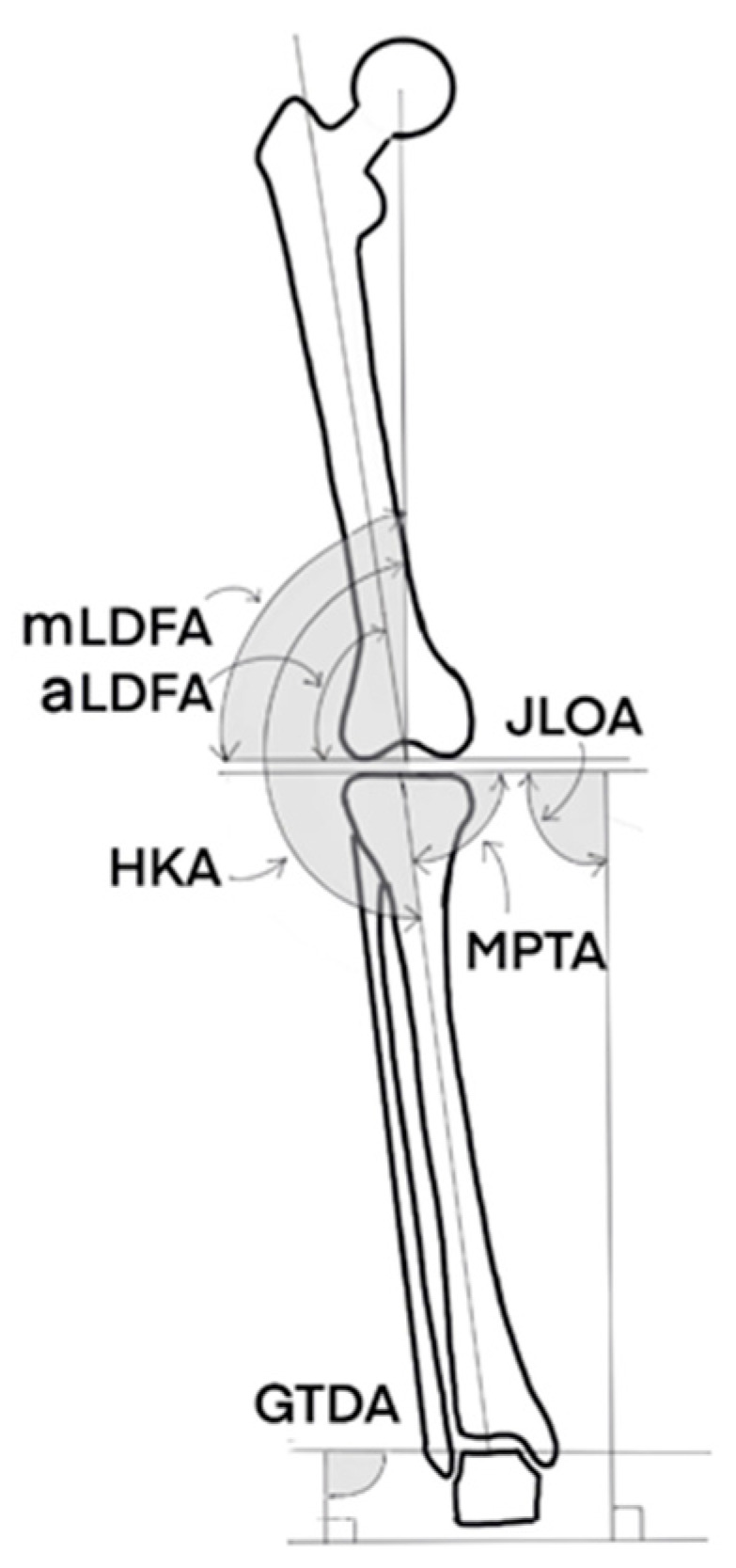
Measurements of the different radiographic angles. HKA: Hip-Knee-Ankle angle. mLDFA: mechanical Lateral Distal Femoral Angle. aLDFA: anatomic Lateral Distal Femoral Angle. MPTA: Medial Proximal Tibial Angle. JLOA: Joint Line Obliquity Angle. GTDA: Ground Talar Dome Angle.

Radiographic: A single observer (ESM) measured the different radiographic alignment parameters (Figure 1) using an image-analysis software (eUnity, Mach7 Technologies, Burlington, VT, USA). All patients obtained pre- and post-operative full weight-bearing long-leg standing radiographs (LLR) according to a standardized protocol. Each patient was barefoot and placed their feet together with the patellae oriented forward to avoid rotational variation [16]. The Hip-Knee-Ankle angle (HKA) was defined as the angle between a line connecting the centers of the femoral head and knee and a line connecting the centers of the knee and the center of the talar dome in the ankle on the LLR (+ varus, —valgus). The mechanical lateral distal femoral angle (mLDFA) was the angle between a line tangent to the distal femoral joint/component and a line connecting the centers of the femoral head and knee (>90 varus, <90 valgus). The anatomic Lateral Distal Femoral Angle (aLDFA) was defined as the angle subtended between a line tangent to the distal femoral joint/component and a line connecting the piriformis fossa and the center of the distal femur (>90 varus, <90 valgus). The medial proximal tibial angle (MPTA) was the angle between a line tangent to the proximal tibial plateau/component and a line connecting the centers of the knee and ankle on the LLR (<90 varus, >90 valgus). The joint line obliquity angle (JLOA) was defined as the angle between the tibial plateau axis and the floor, with positive JLOA values assigned to a medially down-sloping tibial plateau axis. The ground talar dome angle (GTDA) was defined as the angle between the talar dome and the floor, with positive GTDA values assigned to a laterally down-sloping talar dome axis [17] (Figure 1).

### 2.3. Surgical Technique

The goal prior to surgery was to determine what the patient’s native coronal anatomy before the primary TKA surgery and what adjustments would be required to restore it using KA principles. The detailed technique has already been described [9]. In summary, two reference planes are needed to reverse-engineer the patient’s native coronal bone anatomy: the native tibial joint line angle (MPTA) and the anatomic lateral distal femoral angle (aLDFA). Both are measured from the anatomic axis of the long bone which can therefore be used intraoperatively to establish the reference plane. These values need to be measured on both preoperative long leg X-rays (or scout CT scan) showing the patient’s native, pre-operative anatomy and images of the patient’s existing TKA. In the absence of preoperative images for reference, the patient’s contralateral knee can often provide an adequate set of surrogate measurements or a less arthritic version of the knee in question [18].

Following removal of all components. correcting the tibial cut back to native alignment is the priority. As non-anatomic MA cuts in native varus knees tend to resect more bone laterally than medially, the cutting bloc is positioned so that there is no bone cut from the lateral tibial margin of the tibia and that as much bone as necessary is resected on the medial side to restore the native MPTA. This is usually best done with an extramedullary guide referencing the tibial anatomic axis to the center of the talus. Slope is generally decreased to < 5 degrees to accommodate PS revision knees. Once the tibial cut is performed and checked, the distal femoral cut is performed in sufficient valgus to match the new tibial varus and create a balanced extension gap. The femoral cut is anchored to the existing medial cut and any additional bone is resected from the lateral side. Finally, as most MA surgeons set the femoral component in 3 degrees of external rotation from the posterior condylar axis, the femoral rotation of the revised component must be internally rotated at least 3 degrees from where the components were previously seated. In flexion, we aim to obtain 1 to 3 mm of lateral opening at 90 degrees of flexion while maintaining a stable medial compartment. Often a medial augment is required to maintain rotation. If stems are required, short, small diameter cemented stems are selected to enable non-axial placement in the femur and tibia. If necessary, cones are used to support the shorter stems.

Intraoperatively, the net result should be a knee that is stable with a standard, non-constrained polyethylene insert, no play at all in extension, a medial joint line that is stable throughout the arc of motion (medial pivot) and, on the lateral side, a gap that starts balanced in extension and gradually increases to 2–4 mm of laxity at 90°. The patella will track centrally.

### 2.4. Prosthesis

When both femoral and tibial components were revised, the implant used was the Triathlon TS Knee System (Stryker Orthopedics, Mahwah, NJ, USA). Cemented femoral and tibial stems were used in different lengths 50 mm—(short), and 100 mm—(medium) lengths. The stems have a fixed angle of 6◦ of valgus to the femoral component and neutral alignment to the tibial component. Femoral and metaphyseal cones are available. When revising only one component, the appropriate matching device was used following the same principles (short, cemented stems with or without cones).

### 2.5. Statistical Analysis

Statistical analysis was performed using the JMP^®^ Pro software 16.0.0 version (www.jmp.com, SAS, Cary, NC, USA; accessed on 1 October 2022). Continuous data are presented using mean, standard deviation, minimum and maximum values. Comparisons of the native, pre-revision and post-revision continuous data were analyzed using a paired T test (native, pre-, and post-revision for mLDFA, MPTA, JLOA and GTDA). Significance was set at *p* < 0.05. As this is a case report with no comparison group, no sample size calculation was performed. 

## 3. Results

The average age of the seven patients included was 70 ± 9 years and 4 were females. The pre-revision clinical characteristics are listed in Table 1. The mean time between the primary TKA and the revision TKA was 42 ± 27 months.

Three patients were revised for MFI and 4 for stiffness. For five patients both femoral and tibial components were revised, and for the remaining two patients only the femoral component was revised. When both components were revised, a short tibial cemented stem (50 mm) was used in three cases and a medium tibial length stem (100 mm) was used in one case, and a metaphyseal tibial cone was used for one case. Short (50 mm) and medium length (100 mm) cemented stems were used for the femur for four and three cases, respectively. A revision stemmed posterior stabilized femoral component and posterior stabilized insert was used in all cases.

Mean follow-up was 32 ± 26 months (range 13–70). The mean gain of KSS between pre- and post-revision was 75 ± 27 points (range 44–122) (*p* < 0.001). The mean maximum flexion gain was 30° ± 23° (range 5–65) (*p* = 0.01). In all cases, the pain score was reported as improved compared to the pre-revision score (2.3 ± 1.8 vs. 6.6 ± 0.8; *p* < 0.001, respectively). Three patients reported being satisfied and four were very satisfied with their revision procedure. No complications were observed during follow-up.

**Figure 2:**
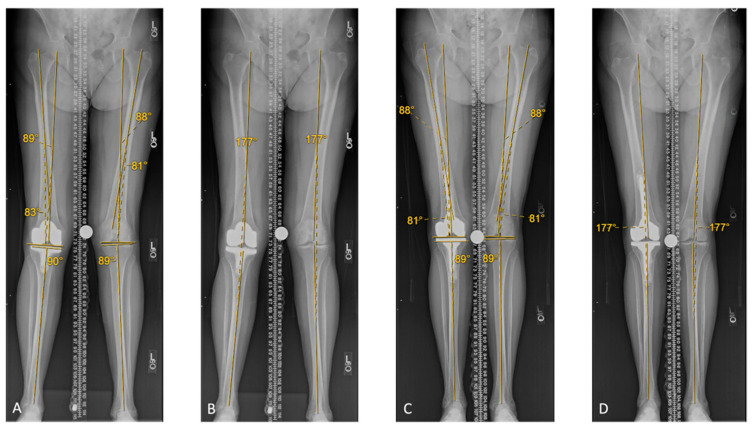
Case study: 80 year old female patient revised for pain and stiffness (20–90°). (**A**) Left (native) leg: imaging of the contralateral knee demonstrates a MPTA angle of 1 degrees of varus, and an aLDFA angle of 9 degrees of valgus. Right (operated) leg: the MPTA was neutral (1 degree variance from native), and the aLDFA was 7 degrees of valgus (2 degrees variance from native). (**B**) Left (native) leg: Scanogram shows a 3 degree valgus HKA and −5 degree GTDA (not shown). Right (operated) leg: Scanogram shows a 3 degree valgus HKA and −19 degree GTDA (not shown). (**C**) Right leg (operated): following KA rTKA, the MPTA is 1 degree of varus, and the aLDFA is 9 degrees of valgus. Following revision, the MPTA, aLDFA, were restored. (**D**) Right leg (operated): scanogram shows a 3 degree valgus HKA angle. The GTDA (not shown) was restored at −5 degrees. At 1 year follow-up, her range of motion was 0–115°.

Native, pre-revision and post-revision limb alignments are summarized in Table 2. After the revision procedure, all radiological limb alignment measurements were restored within 2 degrees relative to the native knee alignment for all patients. No radiolucent lines nor osteolysis were noted on radiographic evaluation. One case is illustrated in Figure 2.

## 4. Discussion

The main finding of this preliminary report is that in 7 highly selected patients rTKA performed using KA principles as described restored the native coronal joint line orientation angles as well as the native overall limb alignment with reasonable accuracy. Furthermore, restoring normal anatomy in these patients successfully resolved MFI, improved range of motion, and increased clinical outcomes scores in all patients. No complications nor revision surgeries were noted within the short- to mid-term follow-up of this small cohort.

With respect to the efficacy of reverse engineering coronal limb alignment and joint line orientation, our data suggests that the native limb alignment successfully restored all angular measures of alignment within 2 degrees for all patients. Femoral component rotation was not measured. Several studies [19,20,21,22] have shown that KA allows for a better restoration of the JLOA compared to MA which corroborates the findings of the present report. Furthermore, Kim et al. [23] found that postoperative ankle joint line orientation after KA primary TKA was horizontal to the floor and closer to that of native ankle joints than those after MA primary TKA. This is in line with the present report where no significant differences of the ankle joint lines were observed between the native and postoperative knees following restoration of native alignment, whereas a significant difference of the GTDA was observed between the native and postoperative MA primary TKA.

With respect to resolving stiffness and MFI, our report suggests that revision into KA resolves these complications, at least in part. rTKA performed for stiffness using traditional techniques is complicated by recurrence of stiffness with reported rates between 7.1% and 49% [1,24,25,26,27,28]. In all these prior studies, however, revision TKA was performed according to MA principles. It stands to reason that if the revision puts the new implants in the same position as the prior components, the procedure is not likely to achieve a different result. In the present report, revising MA TKAs that failed due to stiffness or MFI using KA principles resolved stiffness and allowed a mean maximum flexion exceeding 110 degrees which is rarely obtained in the literature after rTKA [26]. We suggest that a more physiological knee balance is restored after KA rTKA than MA rTKA as the range of motion follows the native axis of rotation and avoids overstuffing of the lateral flexion gap. Notably, however, the ROM is still less than one would expect from a KA primary TKA.

We report being able to restore balance without the use of semi constrained inserts in all knees. Kostretzis et al. reporting results after rTKA using restricted KA [14] found that in contrast to other studies using the Triathlon TS revision Knee System where TS inserts were used almost systematically in MA rTKA, the restricted KA rTKA protocol they used allowed most of the cases to be balanced with a standard PS insert (72%). 

This report has several limitations. First, the authors were inherently limited by the retrospective nature of the report. Second, the number of patients included was limited by the highly restrictive inclusion criteria intended to identify patients with poor outcomes following MA TKAs. Third, the follow-up is short and longer-term follow-up is necessary to assess implant survivorship. However, 15 years following the introduction of KA principles, aseptic loosening has not surfaced as a complication of this technique [13,29,30]. Fourth, femoral component rotation was not assessed and would require three-dimension images such as a CT scan to measure and we cannot comment on our ability to restore normal femoral rotation. However, the lack of flexion instability in these TKAs despite unconstrained inserts suggests that knee’s rotational axis was restored. Fifth, KA was first described for CR implants but in revision surgery PS components are required for the use of stems. Good results have been reported for KA using PS knees [20] but more data needs to be published in this regard. Lastly, existing instrumentation is not designed to facilitate anything other than MA. The reverse-engineering technique described in this article to restore native anatomy remains difficult to perform. Lastly, we report results that are far better than those reported for MA rTKA but acknowledge that, at short term follow up, we were not able to restore the type of motion one could expected from a primary KA TKA which is reported to be nearly physiologic.

This study is clinically relevant as it indirectly proves the thesis that stiffness and MFI following MA TKA is caused by the non-anatomic alignment of the components. Correcting alignment back to native relationships through revisions performed using KA principles can resolve both stiffness and mid-flexion instability.

## 5. Conclusions

In a limited series of patients, revision of stiff or unstable TKA from MA to KA resulted in improved range of motion by 30 degrees on averages, resolved instability without the use of constrained liners, improved clinical outcomes with a mean gain of 75 points on the KSS, and restored constitutional limb alignment within 2 degrees in all patients. As these short term results are promising, further study is warranted.

## Figures and Tables

**Table 1:** Patients pre-revision demographics, knee range of motion, and functional scores for the 7 patients with primary failed-total knee arthroplasty.

Characteristics	Pre-Revision Means ± SD (Range)
Body-Mass-Index	27 ± 3 kg/m^2^ (22 to 31)
Extension	10 ± 8° (0 to 20°)
Flexion	84 ± 35° (40 to 125°)
Knee Society Score Knee (100 is best, 0 is worst)	47 ± 7 points (38 to 56)
Knee Society Score Function (100 is best, 0 is worst)	49 ± 20 points (25 to 75)
Total Knee Society Score (200 is best, 0 is worst)	96 ± 23 points (69 to 131)

**Table 2:** Pre-operative, pre-revision TKA and post-revision TKA radiological outcomes.

Radiological Measurements	Native Alignment Means ± SD (Range)	Pre-Revision TKA Means ± SD (Range)	Post-Revision TKA Means ± SD (Range)	*p*-Value
Hip-Knee-Ankle Angle	176 ± 5° (170 to 184°)	181 ± 4° (177 to 183°)	179 ± 2° (176 to 181°)	0.005 * 0.051 **
Mechanical Lateral Distal Femoral Angle	88 ± 3° (83 to 91°)	90 ± 2° (87 to 92°)	88 ± 3° (83 to 91°)	0.03 * 0.5 **
Medial Proximal Tibial Angle	88 ± 2° (85 to 90°)	90 ± 1° (88 to 92°)	88 ± 2° (85 to 90°)	0.04 * 1 **
Joint Line Obliquity Angle	2 ± 3° (−2 to 5°)	−0.3 ± 3° (−5 to 4°)	1 ± 3° (−2 to 5°)	0.03 * 0.2 **
Ground Talar Dome Angle	−1 ± 5° (−7 to 5°)	−7 ± 7° (−19 to −1°)	−2 ± 4° (−6 to 3°)	<0.001 * 0.3 **

* Paired T Test between native and pre-revision condition. ** Paired T Test between native and post-revision condition.

## References

[1] Schmidt A., Batailler C., Lording T., Badet R., Servien E., Lustig S., Writing Committee (2020). Why Reintervention After Total Knee Arthroplasty Fails? A Consecutive Cohort of 1170 Surgeries. J. Arthroplast..

[2] Baker P., Cowling P., Kurtz S., Jameson S., Gregg P., Deehan D. (2012). Reason for Revision Influences Early Patient Outcomes after Aseptic Knee Revision. Clin. Orthop. Relat. Res..

[3] Sheng P.-Y., Konttinen L., Lehto M., Ogino D., Jämsen E., Nevalainen J., Pajamäki J., Halonen P., Konttinen Y.T. (2006). Revision Total Knee Arthroplasty: 1990 through 2002. A Review of the Finnish Arthroplasty Registry. J. Bone Jt. Surg. Am..

[4] Insall J., Scott W.N., Ranawat C.S. (1979). The Total Condylar Knee Prosthesis. A Report of Two Hundred and Twenty Cases. J. Bone Jt. Surg. Am..

[5] Churches T., Naylor J., Harris I.A. (2017). Arthroplasty Clinical Outcomes Registry National (ACORN) Annual Report, 2016.

[6] Nam D., Nunley R.M., Barrack R.L. (2014). Patient Dissatisfaction Following Total Knee Replacement: A Growing Concern?. Bone Jt. J..

[7] Gunaratne R., Pratt D.N., Banda J., Fick D.P., Khan R.J.K., Robertson B.W. (2017). Patient Dissatisfaction Following Total Knee Arthroplasty: A Systematic Review of the Literature. J. Arthroplast..

[8] Hamilton D.F., Simpson P.M., Patton J.T., Howie C.R., Burnett R. (2017). Aseptic Revision Knee Arthroplasty With Total Stabilizer Prostheses Achieves Similar Functional Outcomes to Primary Total Knee Arthroplasty at 2 Years: A Longitudinal Cohort Study. J. Arthroplast..

[9] Howell S.M., Bini S.A., Steele G.D., Howell S.M., Bini S.A., Steele G.D. (2021). Revision Total Knee Arthroplasty Using Kinematic Alignment Principles. Calipered Kinematically Aligned Total Knee Arthroplasty: Theory, Surgical Techniques, Perspectives.

[10] Rivière C., Vigdorchik J.M., Vendittoli P.-A. (2019). Mechanical Alignment: The End of an Era!. Orthop. Traumatol. Surg. Res..

[11] Howell S.M., Bini S.A., Steele G.D., Howell S.M., Bini S.A., Steele G.D. (2021). Calipered Kinematically Aligned Total Knee Arthroplasty Performed with Specific Manual Instrumentation, Verification Checks, and A Decision Tree. Calipered Kinematically Aligned Total Knee Arthroplasty: Theory, Surgical Techniques, Perspectives.

[12] Luo Z., Zhou K., Peng L., Shang Q., Pei F., Zhou Z. (2019). Similar Results with Kinematic and Mechanical Alignment Applied in Total Knee Arthroplasty. Knee Surg. Sports Traumatol. Arthrosc..

[13] Sappey-Marinier E., Pauvert A., Batailler C., Swan J., Cheze L., Servien E., Lustig S. (2020). Kinematic versus Mechanical Alignment for Primary Total Knee Arthroplasty with Minimum 2 Years Follow-up: A Systematic Review. SICOT J..

[14] Kostretzis L., Roby G.B., Martinov S., Kiss M.-O., Barry J., Vendittoli P.-A. (2021). Revision Total Knee Arthroplasty With the Use of Restricted Kinematic Alignment Protocol: Surgical Technique and Initial Results. Front. Surg..

[15] Insall J.N., Dorr L.D., Scott R.D., Scott W.N. (1989). Rationale of the Knee Society Clinical Rating System. Clin. Orthop. Relat. Res..

[16] Paley D., Tetsworth K. (1992). Mechanical Axis Deviation of the Lower Limbs. Preoperative Planning of Uniapical Angular Deformities of the Tibia or Femur. Clin. Orthop. Relat. Res..

[17] Jeong B.O., Kim T.Y., Baek J.H., Jung H., Song S.H. (2018). Following the Correction of Varus Deformity of the Knee through Total Knee Arthroplasty, Significant Compensatory Changes Occur Not Only at the Ankle and Subtalar Joint, but Also at the Foot. Knee Surg. Sports Traumatol. Arthrosc..

[18] MacDessi S.J., Griffiths-Jones W., Harris I.A., Bellemans J., Chen D.B. (2020). The Arithmetic HKA (AHKA) Predicts the Constitutional Alignment of the Arthritic Knee Compared to the Normal Contralateral Knee: A Matched-Pairs Radiographic Study. Bone Jt. Open.

[19] MacDessi S.J., Allom R.J., Griffiths-Jones W., Chen D.B., Wood J.A., Bellemans J. (2022). The Importance of Joint Line Obliquity: A Radiological Analysis of Restricted Boundaries in Normal Knee Phenotypes to Inform Surgical Decision Making in Kinematically Aligned Total Knee Arthroplasty. Knee Surg. Sports Traumatol. Arthrosc..

[20] MacDessi S.J., Griffiths-Jones W., Chen D.B., Griffiths-Jones S., Wood J.A., Diwan A.D., Harris I.A. (2020). Restoring the Constitutional Alignment with a Restrictive Kinematic Protocol Improves Quantitative Soft-Tissue Balance in Total Knee Arthroplasty: A Randomized Controlled Trial. Bone Jt. J..

[21] Sappey-Marinier E., Batailler C., Swan J., Schmidt A., Cheze L., MacDessi S.J., Servien E., Lustig S. (2021). Mechanical Alignment for Primary TKA May Change Both Knee Phenotype and Joint Line Obliquity without Influencing Clinical Outcomes: A Study Comparing Restored and Unrestored Joint Line Obliquity. Knee Surg. Sports Traumatol. Arthrosc..

[22] Tran T., McEwen P., Peng Y., Trivett A., Steele R., Donnelly W., Clark G. (2022). Kinematic Alignment in Total Knee Arthroplasty: A Five-Year Prospective, Multicentre, Survivorship Study. Bone Jt. Open.

[23] Kim J.-T., Han J., Lim S., Shen Q.H., Won Y.Y. (2019). Kinematically Aligned TKA Aligns the Ankle Joint Line Closer to Those of the Native Ankle than Mechanically Aligned TKA in Bipedal Stance. J. Knee Surg..

[24] Moya-Angeler J., Bas M.A., Cooper H.J., Hepinstall M.S., Rodriguez J.A., Scuderi G.R. (2017). Revision Arthroplasty for the Management of Stiffness After Primary TKA. J. Arthroplast..

[25] Kim G.K., Mortazavi S.M.J., Parvizi J., Purtill J.J. (2012). Revision for Stiffness Following TKA: A Predictable Procedure?. Knee.

[26] Cohen J.S., Gu A., Lopez N.S., Park M.S., Fehring K.A., Sculco P.K. (2018). Efficacy of Revision Surgery for the Treatment of Stiffness After Total Knee Arthroplasty: A Systematic Review. J. Arthroplast..

[27] Christensen C.P., Crawford J.J., Olin M.D., Vail T.P. (2002). Revision of the Stiff Total Knee Arthroplasty. J. Arthroplast..

[28] Heesterbeek P.J.C., Goosen J.H.M., Schimmel J.J.P., Defoort K.C., van Hellemondt G.G., Wymenga A.B. (2016). Moderate Clinical Improvement after Revision Arthroplasty of the Severely Stiff Knee. Knee Surg. Sports Traumatol. Arthrosc..

[29] Howell S.M., Shelton T.J., Hull M.L. (2018). Implant Survival and Function Ten Years After Kinematically Aligned Total Knee Arthroplasty. J. Arthroplast..

[30] Lee Y.S., Howell S.M., Won Y.-Y., Lee O.-S., Lee S.H., Vahedi H., Teo S.H. (2017). Kinematic Alignment Is a Possible Alternative to Mechanical Alignment in Total Knee Arthroplasty. Knee Surg. Sports Traumatol. Arthrosc..

